# Serum Lactate Level in Early Stage Is Associated With Acute Kidney Injury in Traumatic Brain Injury Patients

**DOI:** 10.3389/fsurg.2021.761166

**Published:** 2022-01-31

**Authors:** Ruoran Wang, Shaobo Wang, Jing Zhang, Min He, Jianguo Xu

**Affiliations:** ^1^Department of Neurosurgery, West China Hospital, Sichuan University, Chengdu, China; ^2^Department of Infectious Diseases, Xi'an Hospital of Traditional Chinese Medicine, Xi'an, China; ^3^Department of Critical Care Medicine, West China Hospital, Sichuan University, Chengdu, China

**Keywords:** serum lactate, acute kidney injury, traumatic brain injury, marker, predictive model

## Abstract

**Background:**

Acute kidney injury (AKI) is a common complication in the clinical practice of managing patients with traumatic brain injury (TBI). Avoiding the development of AKI is beneficial for the prognosis of patients with TBI. We designed this study to testify whether serum lactate could be used as a predictive marker of AKI in patients with TBI.

**Materials and Methods:**

In total, 243 patients with TBI admitted to our hospital were included in this study. Univariate and multivariate logistic regression analyses were utilized to analyze the association between lactate and AKI. The receiver operating characteristic (ROC) curves were drawn to verify the predictive value of lactate and the logistic model.

**Results:**

Acute kidney injury group had higher age (*p* = 0.016), serum creatinine (*p* < 0.001), lactate (*p* < 0.001), and lower Glasgow Coma Scale (GCS; *p* = 0.021) than non-AKI group. Multivariate logistic regression showed that age [odds ratio (OR) = 1.026, *p* = 0.022], serum creatinine (OR = 1.020, *p* = 0.010), lactate (OR = 1.227, *p* = 0.031), fresh frozen plasma (FFP) transfusion (OR = 2.421, *p* = 0.045), and platelet transfusion (OR = 5.502, *p* = 0.044) were risk factors of AKI in patients with TBI. The area under the ROC curve (AUC) values of single lactate and predictive model were 0.740 and 0.807, respectively.

**Conclusion:**

Serum lactate level in the early phase is associated with AKI in patients with TBI. Lactate is valuable for clinicians to evaluate the probability of AKI in patients with TBI.

## Introduction

Traumatic brain injury (TBI) is a public health issue, which has been paid widespread attention for the high incidence of mortality and disability. In addition to the initial brain injury severity, complications of the extracranial organ after injury also play a significant role in the poor prognosis of patients with TBI (1, 2). Among those organ dysfunctions, acute kidney injury (AKI) has received much attention for the high prevalence and close correlation with prognosis in patients with TBI. There are still no effective pharmaceuticals to treat and alleviate the unfavorable effects of AKI. Therefore, discovering risk factors of AKI in patients with TBI in the early phase and evaluating the possibility of developing AKI among them are essential to avoid the occurrence of AKI by reducing the usage of nephrotoxic drugs and adjusting treatment strategies. Recently, some research studies explored the value of neutrophil gelatinase-associated lipocalin (NGAL), kidney injury molecule-1 (KIM-1), and insulin-like growth factor-binding protein-7 (IGFBP-7) in evaluating the risk of AKI and even diagnosing AKI among critically ill patients, such as TBI (3–10). However, the results of these studies showed different values of these markers in predicting AKI. Additionally, high costs and no clear clinical interpretation of these markers make them still unable to be conveniently used in current clinical practice. While discovering some readily available markers and utilizing them to assess the risk of AKI are more available and practical for clinicians under conditions of limited resources.

As a commonly and conveniently used marker, the serum lactate level, which is usually considered as the reflect of tissue hypoperfusion, has been verified associated with mortality in many kinds of patients in previous studies (11–18). However, only a few studies confirmed the value of serum lactate in predicting AKI among specific patients (19–24). This study explores whether serum lactate level in the early phase after TBI is associated with AKI and verifies its ability for predicting AKI.

## Materials and Methods

### Study Patients

**Figure 1:**
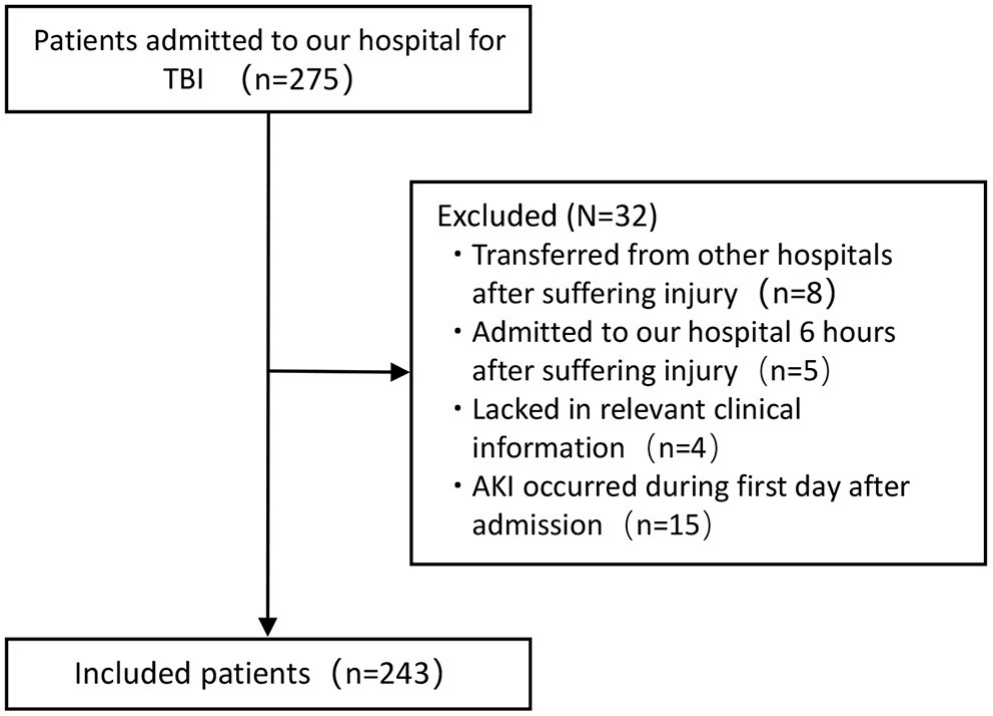
Flowchart of patients inclusion.

Patients admitted to the emergency department of West China hospital for TBI between September 2016 and June 2019 were eligible for this study. The diagnosis of TBI was confirmed by injury signs of CT or MRI of the head region after admission. Patients were excluded based on the following criteria: (1) patients transferred from other hospitals after suffering injury; (2) patients admitted to our hospital 6 h after suffering from injury; (3) patients lacked in relevant clinical information; (4) patients had developed AKI during the first day after admission. The flowchart of inclusion is shown in Figure 1. In total, 243 patients were finally included in this study. This study was approved by the ethics committee of West China hospital and performed abiding by the Declaration of Helsinki. Informed consent forms about joining the observational study of each patient were routinely signed by patients themselves or their legally authorized representatives in our hospital.

### Data Collection

Demographic information and clinical information, such as injury mechanism, vital signs on admission, Glasgow Coma Scale (GCS) on admission, injury severity score (ISS), laboratory tests, radiologic signs surgical options, records of transfusion during the first 24 h after admission, and abbreviated injury score (AIS) of head, chest, abdomen, and limbs, were collected. Results of laboratory tests were acquired by analyzing the first blood sample collected once patients were admitted to the emergency department of our hospital. The outcome of this study was the development of AKI on the second day after admission. Moreover, AKI was diagnosed according to the Kidney Disease Improving Global Outcome (KDIGO) criteria.

### Statistical Analysis

The normality of included variables was testified using the Kolmogorov-Smirnov test. Normally distributed and non-normally distributed variables were presented as mean ± SD and median (interquartile range), respectively. Categorical variables were shown in the form of numbers (percentage). We performed Independent Student's *t*-test and the Mann-Whitney U test, respectively, to analyze the difference between two groups of normally distributed and non-normally distributed variables. A χ^2^ test or Fisher test was utilized to compare the difference of categorical variables. Univariate logistic regression analysis was firstly performed to explore potential risk factors of AKI. Then, significant factors in univariate logistic regression with *p* < 0.05 were included in multivariate logistic regression analysis with the stepwise forward method. Finally, factors still significant in multivariate analysis were incorporated to construct a logistic model to predict the development of AKI. Nomogram was drawn to visually show the predictive model. The receiver operating characteristic (ROC) curves were drawn, and the area under the ROC curve (AUC) was calculated to assess the predictive value of single lactate and the logistic model.

A two-sided value of *p* < 0.05 was considered to be statistically significant. SPSS 22.0 Windows software (SPSS, Inc., Chicago, IL, USA) and R (version 3.6.1; R Foundation) were used for all statistical analyses.

## Results

### Baseline Characteristic of Included Patients

Among overall included patients, 34 developed AKI with an incidence of 14.0% (Table 1). In the AKI group, the median of time from admission to AKI and AKI stage was 2 days and stage 1. AKI group had higher age (49 vs. 40, *p* = 0.016), serum creatinine (90 vs. 66, *p* < 0.001), lactate (3.9 vs. 2.1, *p* < 0.001), and lower GCS (5 vs. 6, *p* = 0.021) than non-AKI group. Compared with non-AKI group, AKI group was more likely to receive fresh frozen plasma (FFP) transfusion (55.9% vs. 23.4%, *p* < 0.001) and platelet transfusion (11.8% vs. 1.4%, *p* = 0.008). The mortality of the AKI group was significantly higher than that of the non-AKI group; while the length of ICU stay (4 vs. 12, *p* = 0.027) and the length of hospital stay (8 vs. 21, *p* = 0.001) were significantly shorter in the AKI group.

**Table 1:** Baseline characteristics of included TBI patients with and without AKI.

**Characteristics**	**Overall patients (*N* = 243)**	**Non-AKI patients (*n* = 209, 86.0%)**	**AKI patients (*n* = 34, 14.0%)**	***P***
Age (year)	41 (26–55)	40 (26–53)	49 (33–67)	0.016
Male	183 (75.3%)	155 (74.2%)	28 (82.4%)	0.289
**Injury mechanism**				0.048
Motor vehicle accident	149 (61.3%)	131 (62.7%)	18 (52.9%)	
Falling injury	51 (21.0%)	42 (20.1%)	9 (26.5%)	
Stumble	28 (11.5%)	21 (10.0%)	7 (20.6%)	
Others	15 (6.2%)	15 (7.2%)	0	
Prehospital time (h)	1 (1–2)	1 (1–2)	1 (1–2)	0.772
**Vital signs on admission**				
Systolic blood pressure (mmHg)	122 (106–139)	121 (106–138)	124 (105–151)	0.455
Diastolic blood pressure (mmHg)	73 ± 16.56	73 ± 15.51	73 ± 22.24	0.905
Heart rate (bpm)	100 (82–120)	100 (82–120)	96 (73–109)	0.120
Body temperature (°C)	36.8 (36.5–37.0)	36.8 (36.5–37.2)	36.8 (36.4–37.0)	0.588
Anisocoria	91 (37.4%)	80 (38.3%)	11 (32.4%)	0.504
GCS	5 (4–7)	6 (4–7)	5 (3–6)	0.021
AIS head	5 (4–5)	4 (4–5)	5 (4–5)	0.068
AIS chest	0 (0–0)	0 (0–0)	0 (0–3)	0.138
AIS abdomen	0 (0–0)	0 (0–0)	0 (0–0)	0.835
AIS limbs	0 (0–0)	0 (0–0)	0 (0–0)	0.837
ISS	25 (16–25)	25 (16–25)	25 (16–34)	0.083
**Laboratory tests**				
White blood cell (109/L)	15.73 (11.48–20.10)	15.45 (11.33–19.47)	15.96 (13.05–21.42)	0.197
Hemoglobin (g/L)	87 (75–106)	89 (75–108)	82 (74–89)	0.133
Lactate dehydrogenase (U/L)	373 (294–514)	370 (294–502)	382 (289–554)	0.856
Serum Lactate (mmol/L)	2.3 (1.5–3.6)	2.1 (1.4–3.2)	3.9 (2.6–5.5)	<0.001
Serum creatinine (umol/L)	69 (53–89)	66 (51–84)	90 (69–116)	<0.001
**Radiologic signs**				
Epidural hematoma	25 (10.3%)	23 (11.0%)	2 (5.9%)	0.545
Subdural hematoma	64 (26.3%)	54 (25.8%)	10 (29.4%)	0.664
Subarachnoid hemorrhage	153 (63.0%)	132 (63.2%)	21 (61.8%)	0.876
Diffuse axonal injury	66 (27.2%)	57 (27.3%)	9 (26.5%)	0.922
**Treatments**				
FFP transfusion	68 (28.0%)	49 (23.4%)	19 (55.9%)	<0.001
Fibrinogen transfusion	31 (12.8%)	26 (12.4%)	5 (14.7%)	0.781
Platelet transfusion	7 (2.9%)	3 (1.4%)	4 (11.8%)	0.008
Hemoglobin transfusion	39 (16.0%)	34 (16.3%)	5 (14.7%)	0.816
Decompressive craniectomy	91 (37.4%)	76 (36.4%)	15 (44.1%)	0.390
Hematoma evacuation	103 (42.4%)	90 (43.1%)	13 (38.2%)	0.596
Time from admission to AKI (day)	–	–	2 (2–4)	
AKI stage	–	–	1 (1–2)	
Mortality	123 (50.6%)	96 (45.9%)	27 (79.4%)	<0.001
Length of ICU stay (day)	11 (2–24)	12 (3–25)	4 (2–19)	0.027
Length of hospital stay (day)	18 (5–34)	21 (6–38)	8 (3–22)	0.001

*AKI, acute kidney injury; GCS, Glasgow Coma Scale; AIS, Abbreviated Injury Score; ISS, Injury Severity Score; FFP, fresh frozen plasma*.

### Univariate and Multivariate Logistic Regression Analyses for Risk Factors of AKI in Included Patients With TBI

Results of univariate logistic regression showed that age (*p* = 0.008), GCS (*p* = 0.045), ISS (*p* = 0.043), serum creatinine (*p* < 0.001), lactate (*p* < 0.001), FFP transfusion (*p* < 0.001), and platelet transfusion (*p* = 0.005) were associated with AKI in patients with TBI (Table 2). After adjusting confounding effects, multivariate logistic regression presented that only age [odds ratio (OR) = 1.026, *p* = 0.022], serum creatinine (OR = 1.020, *p* = 0.010), lactate (OR = 1.227, *p* = 0.031), fresh frozen plasma transfusion (OR = 2.421, *p* = 0.045), and platelet transfusion (OR = 5.502, *p* = 0.044) were independent risk factors of AKI in included patients with TBI.

**Table 2:** Univariate and multivariate logistic regression analyses for risk factors of AKI in included TBI patients.

	**Univariate analysis**			**Multivariate analysis**		
**Characteristics**	**OR**	**95%Cl**	***P***	**OR**	**95%Cl**	***P***
Age (year)	1.027	1.007–1.046	**0.008**	1.026	1.004–1.049	**0.022**
Male	1.626	0.639–4.139	0.308			
**Injury mechanism**			0.338			
Motor vehicle accident	1	Reference				
Falling injury	1.560	0.652–3.731	0.318			
Stumble	2.426	0.904–6.510	0.078			
Others	0	0	0.999			
Prehospital time			0.764			
Systolic blood pressure	1.000	0.996–1.005	0.952			
Diastolic blood pressure	1.002	0.980–1.024	0.888			
Heart rate (bpm)	0.989	0.975–1.003	0.120			
Body temperature	0.776	0.502–1.198	0.252			
Anisocoria	0.771	0.357–1.667	0.509			
GCS	0.838	0.704–0.996	**0.045**			
AIS head	1.400	0.867–2.260	0.169			
AIS chest	1.255	0.978–1.610	0.074			
AIS abdomen	0.878	0.440–1.751	0.711			
AIS limbs	1.099	0.709–1.705	0.672			
ISS	1.041	1.001–1.083	**0.043**			
White blood cell	1.034	0.982–1.088	0.207			
Hemoglobin	0.993	0.978–1.009	0.397			
Lactate dehydrogenase	1.000	0.999–1.001	0.939			
Serum Lactate	1.357	1.169–1.575	**<0.001**	1.227	1.019–1.476	**0.031**
Serum creatinine	1.033	1.019–1.046	**<0.001**	1.020	1.005–1.035	**0.010**
Epidural hematoma	0.505	0.114–2.249	0.370			
Subdural hematoma	1.196	0.537–2.662	0.661			
Subarachnoid hemorrhage	0.942	0.447–1.988	0.876			
Diffuse axonal injury	0.960	0.423–2.181	0.922			
FFP transfusion	5.275	2.462–11.303	**<0.001**	2.421	1.021–5.739	**0.045**
Fibrinogen transfusion	1.214	0.431–3.413	0.714			
Platelet transfusion	9.156	1.953–42.928	**0.005**	5.502	1.049–28.846	**0.044**
Hemoglobin transfusion	0.887	0.321–2.455	0.818			
Decompressive craniectomy	1.384	0.670–2.858	0.380			
Hematoma evacuation	0.853	0.409–1.783	0.673			

*OR, odds ratio; CI, confidence interval; GCS, Glasgow Coma Scale; AIS, Abbreviated Injury Score; ISS, Injury Severity Score; FFP, fresh frozen plasma. Bold values mean p < 0.05*.

### Predictive Value of Single Lactate and the Logistic Model

**Figure 2:**
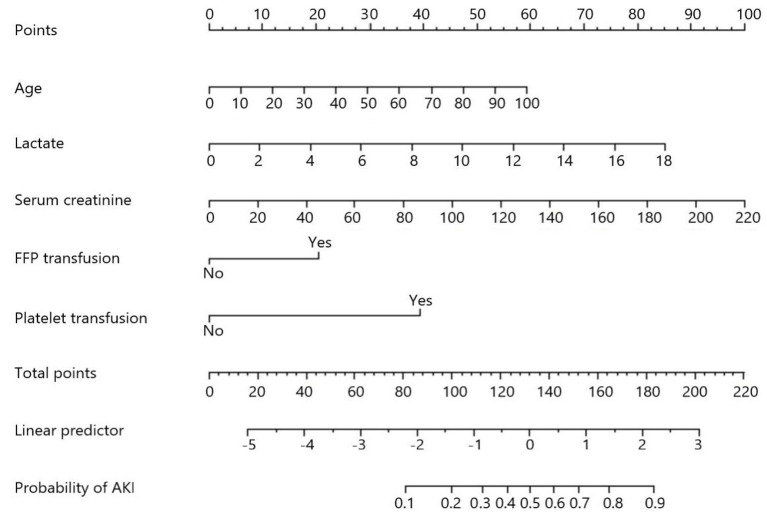
Nomogram of constructed a model for predicting acute kidney injury in included in patients with TBI.

**Figure 3:**
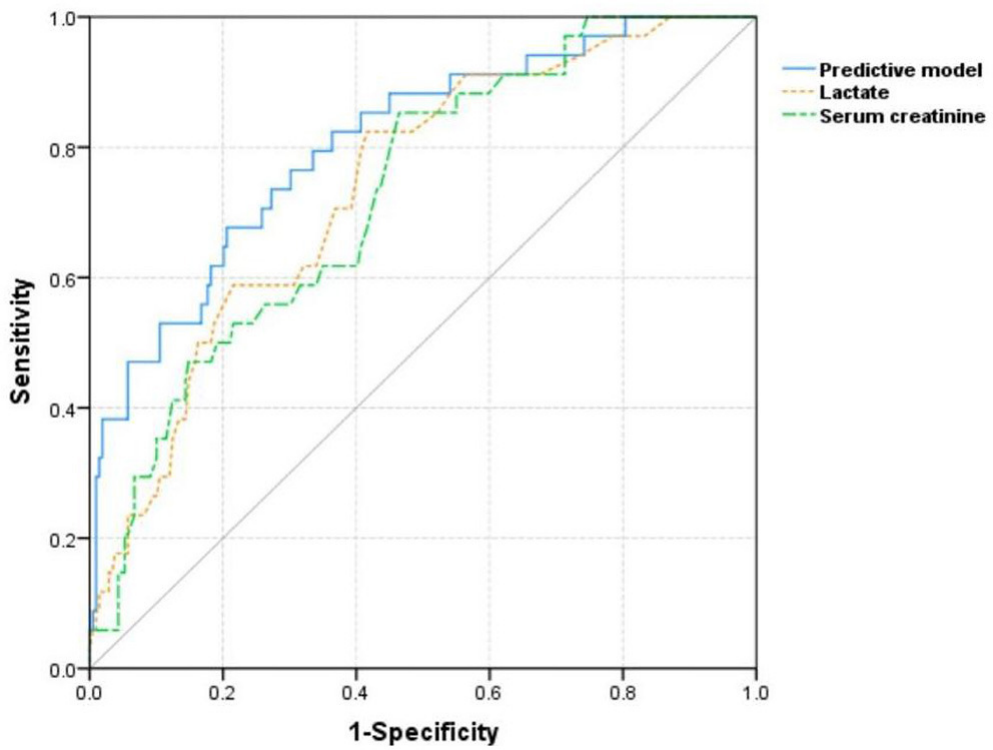
The receiver operating characteristic curves of single lactate and constructed model for predicting acute kidney injury in included patients with TBI.

Five factors significant in multivariate logistic regression that include age, serum creatinine, lactate, fresh frozen plasma transfusion, and platelet transfusion were incorporated to construct a logistic model to predict the development of AKI in patients with TBI. For convenient clinical use, a visual nomogram of this model is drawn and is presented in Figure 2. Additionally, the AUC values of single lactate and predictive model were 0.740 and 0.807, respectively (Table 3). The ROC curves of single lactate and predictive model are presented as Figure 3.

**Table 3:** Predictive value of single lactate and the logistic model.

**Variables**	**AUC**	**95%CI**	**Sensitivity**	**Specificity**	**Best cut-off value**
Serum creatinine	0.728	0.644–0.812	0.853	0.536	68.5
Serum lactate	0.740	0.655–0.824	0.824	0.584	2.45
Predictive model	0.807	0.727–0.887	0.676	0.794	0.1485

*AUC, the area under the receiver operating characteristic curve; CI, confidence interval*.

## Discussion

Acute kidney injury is a common complication during the clinical management of TBI patients with incidence ranging from 7.6 to 24% (10, 25–28). In our study, 15 patients had developed AKI during the first day after admission and were consequently excluded from this study for no much need to predict the AKI development. In addition to these 15 patients, 34 patients developed AKI 1 day after admission with the incidence of 14.0%. Results of our study showed that age, serum creatinine, serum lactate, transfusion of FFP, and platelet were risk factors of AKI in included patients with TBI.

Widely recognized as a biomarker of tissue hypoperfusion, the serum lactate would increase in various kinds of critically ill patients. Actually, many studies have indicated the increased level of serum lactate after suffering from TBI and explored the correlation between increased lactate and prognosis of patients with TBI (29–31). The increased level of serum lactate after TBI may mainly be attributable to the systemic tissue hypoperfusion caused by massive blood loss and impaired cardiopulmonary function. Massive blood loss could lead to a decreased level of hemoglobin, which means reduced oxygen-carrying capacity and subsequent systemic hypoperfusion in patients with TBI, though the threshold of red blood cell transfusion specific for patients with TBI has not reached a consensus. Complications of the respiratory and cardiovascular system after an injury, such as neurogenic pulmonary edema, acute lung injury, and neurogenic myocardial dysfunction, are prevalent in patients with TBI. One previous study reported that the incidence of cardiovascular and respiratory failure reached up to 56 and 43% in severe patients with TBI, respectively (32). These cardiopulmonary dysfunctions could lead to the inadequate gas exchange and unstable cardiac output which may subsequently cause insufficient oxygen supplement in systemic organs and increased anaerobic metabolism. Additionally, the activated renin angiotensin system (RAS) and increased oxidative stress activity in systemic organs after TBI may also result in renal tissue hypoxia through vasoconstriction induced decreased perfusion and increasing oxygen consumption caused by hyperactive mitochondrial function (33–35). The hypoxia has been verified associated with the development of AKI in clinical research studies (36, 37). Moreover, animal experiments also indicated renal hypoxia played a pivotal role in the pathophysiological process of AKI and progression from AKI to chronic kidney disease (38, 39). In summary, hypoperfusion and ischemia of systemic tissues reflected by increased lactate level after TBI could result in the development of an impaired renal function.

In addition to serum lactate, age, initial serum creatinine, FFP transfusion, and platelet transfusion were also confirmed as risk factors of AKI. Some previous studies also confirmed that older age was positively associated with AKI (40, 41). The relation between increased age and AKI development in patients with TBI may be attributable to physiological changes in renal function and multiple comorbidities with age. In some studies, transfusion of FFP or platelet was confirmed as independent risk factor of AKI (42–46). Massive FFP transfusion may aggravate the impaired renal function through activating inflammatory and immunologic reactions (47, 48). The detailed mechanism of platelet transfusion contributing to AKI development has not been fully understood. Some research studies found platelet concentrates contained bioactive CD40 ligands, which could promote the pro-inflammatory markers release (49, 50). Therefore, platelet transfusion may impair renal function by promoting an unfavorable inflammatory state. Another research indicated platelet transfusion could result in the formation of microthrombi existing in the renal microvascular circulation (50). Combining abovementioned five factors, our constructed logistic model was more efficient in predicting AKI with a higher AUC value than single lactate.

This study had several limitations. Firstly, this study was conducted in a single medical center with a relatively inadequate sample size, so that selection bias may not be avoided. The predictive value of our constructed logistic model should be verified in other medical centers. Secondly, the detailed doses of blood products transfusion were not recorded so that we could not accurately analyze the dose relationship between transfusion and AKI. Thirdly, a history of underlying diseases was not included because of the rare incidence of comorbidities in our included patients. Future research studies could be conducted to adjust the confounding effects of comorbidities and investigate whether incorporating comorbidities into our model could improve predictive stability and accuracy. Finally, the serum lactate level was only recorded from analyzing the first blood sample collected on admission. Therefore, we could not analyze the association between AKI and the continuous and average level of serum lactate during a specific time period. Future studies collecting multiple measurements of lactate level within a specific duration are worthy to be designed and conducted to achieve this aim.

## Conclusion

Higher serum lactate level in the early phase is positively associated with the development of AKI in patients with TBI. Measuring lactate is beneficial for clinicians to evaluate the probability of AKI occurrence in patients with TBI.

## Data Availability Statement

The raw data supporting the conclusions of this article will be made available by the authors, without undue reservation.

## Ethics Statement

The studies involving human participants were reviewed and approved by the Ethics Committee of West China Hospital, Sichuan University. Written informed consent to participate in this study was provided by the participants or their legal guardian/next of kin.

## Author Contributions

RW: contributed to the manuscript conception, design, and writing. MH and JZ: contributed to the collection and assembly of data. RW and SW: contributed to the data analysis and interpretation. JX: contributed to the manuscript proofreading and revision. All the authors contributed to the approval of the final manuscript and its publication.

## Funding

This work was supported by the 1.3.5 project for disciplines of excellence, West China Hospital, Sichuan University (ZYJC18007) and Key Research and Development project of the Science and Technology Department of Sichuan Province (2019YFS0392).

## Conflict of Interest

The authors declare that the research was conducted in the absence of any commercial or financial relationships that could be construed as a potential conflict of interest.

## Publisher's Note

All claims expressed in this article are solely those of the authors and do not necessarily represent those of their affiliated organizations, or those of the publisher, the editors and the reviewers. Any product that may be evaluated in this article, or claim that may be made by its manufacturer, is not guaranteed or endorsed by the publisher.
